# Mechanism of COVID-19 Causing ARDS: Exploring the Possibility of Preventing and Treating SARS-CoV-2

**DOI:** 10.3389/fcimb.2022.931061

**Published:** 2022-06-14

**Authors:** Jiajing Zheng, Jiameng Miao, Rui Guo, Jinhe Guo, Zheng Fan, Xianbin Kong, Rui Gao, Long Yang

**Affiliations:** ^1^ College of Traditional Chinese medicine, Tianjin University of Traditional Chinese Medicine, Tianjin, China; ^2^ Research Center for Infectious Diseases, Tianjin University of Traditional Chinese Medicine, Tianjin, China; ^3^ School of Integrative Medicine, Tianjin University of Traditional Chinese Medicine, Tianjin, China; ^4^ Department of Critical Medicine, The First Affiliated Hospital of Suzhou University, Suzhou, China; ^5^ Institute of Clinical Pharmacology of Xiyuan Hospital, China Academy of Chinese Medical Sciences, Beijing, China

**Keywords:** novel coronavirus, acute respiratory distress syndrome, angiotensin-converting enzyme II protein, cytokine storm, immune response, NF-κB pathway, type I interferon

## Abstract

Novel coronavirus pneumonia (COVID-19) is spreading worldwide, causing great harm and stress to humans. Since patients with novel coronavirus (SARS-CoV-2) have a high probability of developing acute respiratory distress syndrome (ARDS) in severe cases, the pathways through which SARS-CoV-2 causes lung injury have become a major concern in the scientific field. In this paper, we investigate the relationship between SARS-CoV-2 and lung injury and explore the possible mechanisms of COVID-19 in ARDS from the perspectives of angiotensin-converting enzyme 2 protein, cytokine storm, activation of the immune response, triggering of Fas/FasL signaling pathway to promote apoptosis, JAK/STAT pathway, NF-κB pathway, type I interferon, vitamin D, and explore the possibility of prevention and treatment of COVID-19. To explore the possibility of SARS-CoV-2, and to provide new ideas to stop the development of ARDS in COVID-19 patients.

## 1 Preface

The SARS-CoV-2 was first discovered in China and showed a multi-point outbreak worldwide. For a long time, human life safety and health have been facing a great threat, and the COVID-19 pandemic has caused more than 1 million deaths worldwide. Some data show that the mortality rate of COVID-19 infection is 26%, the ICU admission rate is 47%, and the risk rate of ARDS due to COVID-19 is 3.2 (aOR, 3.20; 95% CI: 1.65-6.18; p=0.001) (Khamis et al., 2021). SARS-CoV-2 has received widespread attention. The World Health Organization (WHO) declared it a global epidemic and tentatively named the coronavirus as 2019 novel coronavirus (2019-nCoV), and pneumonia infected by this virus as “COVID-19”, which was officially named by the International Committee on Classification of Viruses as “SARS-CoV-2”.

**Figure 1:**
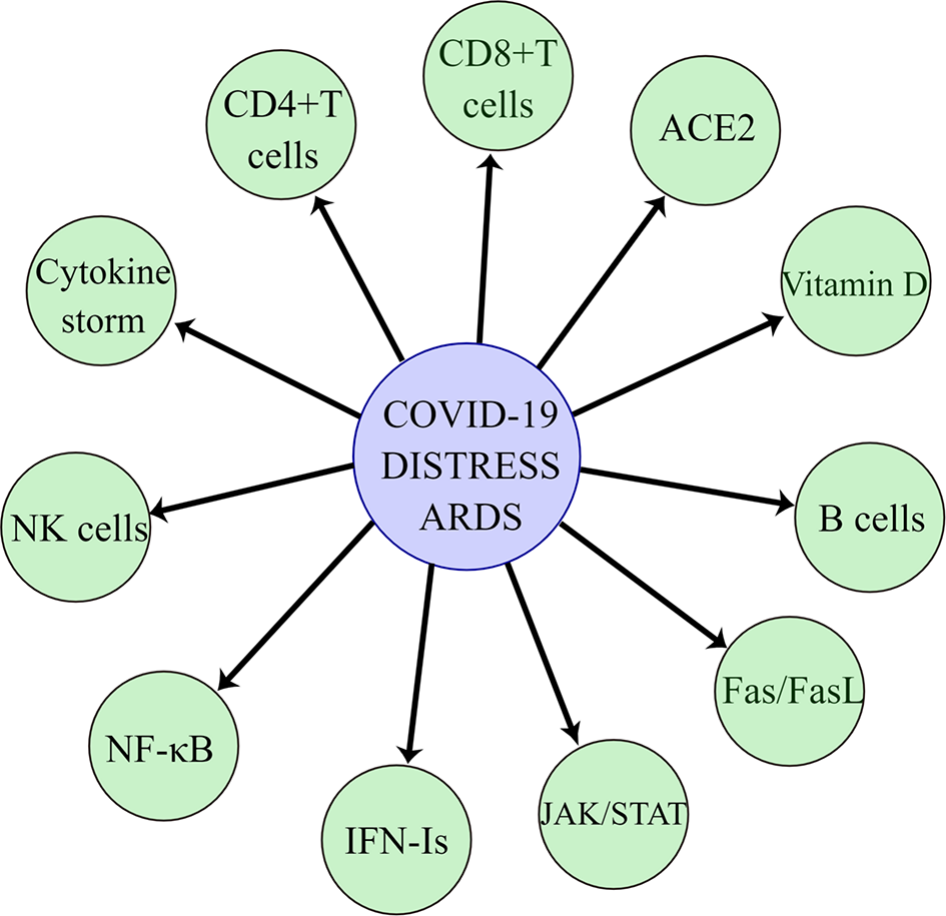
SARS-CoV-2 causes acute respiratory distress syndrome through angiotensin-converting enzyme 2, cytokine storm, immune cells, Fas/FasL pathway, JAK/STAT pathway, NF-κB pathway, type I interferon, and vitamin D.

The most common symptom of COVID-19 disease is fever with coughing, sneezing, weakness, and shortness of breath, which can rapidly develop into acute respiratory distress syndrome in severe cases (Zarrilli et al., 2021). Currently, a large number of studies have been made on the pathogenic mechanism of SARS-CoV-2, but the medical community still has a limited understanding of SARS-CoV-2. Since the SARS-CoV-2 is a highly transmissible and lethal virus and undergoes rapid recombination and mutation in the human body, forming a variety of mutant strains and creating new virulence, this poses a great challenge to humans in the process of antiviral therapy. Understanding the mechanism of lung injury caused by SARS-CoV-2 will best help us find ways to prevent and treat this disease. In this paper, we will discuss the mechanisms of the acute respiratory syndrome caused by SARS-CoV-2 in terms of angiotensin-converting enzyme 2 protein, cytokine storm, activated immune response, triggering Fas/FasL signaling pathway for pro-apoptosis, JAK/STAT pathway, NF-κB pathway, type I interferon, and vitamin D (**Figure 1**).

## 2 Association of SARS-CoV-2 With Acute Respiratory Distress Syndrome

SARS-CoV-2 causes a positive correlation between the incidence and severity of ARDS, and the challenges associated with SARS-CoV-2 and this syndrome are becoming more prominent, for example in terms of their high mortality rate and the lack of effective pharmacological treatment (Meyer et al., 2021). ARDS is characterized histologically by diffuse alveolar damage with increased vascular permeability and reduced compliance, affecting gas exchange and leading to intractable hypoxemia (Batah and Fabro, 2021). Over the last 50 years, researchers have conducted numerous basic and clinical studies on ARDS, but the morbidity and mortality of ARDS remain high and there is a lack of specific drugs for ARDS. Currently, 15-30% of people hospitalized with COVID-19 will go on to develop ARDS (Attaway et al., 2021).

Gibson et al (Gibson et al., 2020) concluded that the prognosis of COVID-19 ARDS appears to be worse than that of ARDS due to other causes, which are pneumonia, pulmonary contusion, toxic substance inhalation, and severe systemic infections. Mortality rates ranged from 26% to 61.5% in patients with COVID-19 ARDS who had been admitted to an intensive care unit, and from 65.7% to 94% in patients receiving mechanical ventilation. Several analyses have shown that COVID-19 ARDS has pathophysiological features similar to those of non-COVID-19 ARDS, namely reduced respiratory compliance, high respiratory mechanics heterogeneity, and hypoxemia. The study suggests that lung-protective ventilation should be implemented in all mechanically ventilated patients with COVID-19 ARDS, and noninvasive ventilation can be performed in mild and moderate patients using dedicated respiratory arrest (Grasselli et al., 2021). Severe COVID-19 produces impairment of ARDS-like hyper inflammation and endothelial dysfunction, ultimately leading to respiratory and multiorgan failure and death. A proportion of surviving patients will have a persistent fibroproliferative response, and interstitial lung disease (ILD) and pulmonary fibrosis will in turn lead to an increased risk of severe disease (Ntatsoulis et al., 2021). This disease is difficult to control in advanced stages, and early treatment is critical for prognosis in controlling and improving symptoms such as pulmonary inflammation, thick airway mucus secretion, elevated levels of pro-inflammatory cytokines, lung injury, and micro thrombosis in patients with ARDS due to SARS-CoV-2 (Quesada-Gomez et al., 2020).

## 3 Possible Mechanisms of the ARDS Caused by SARS-CoV-2

### 3.1 SARS-CoV-2 S Protein Binds to Angiotensin-Converting Enzyme 2 (ACE2) Protein to Cause the ARDS

**Figure 2:**
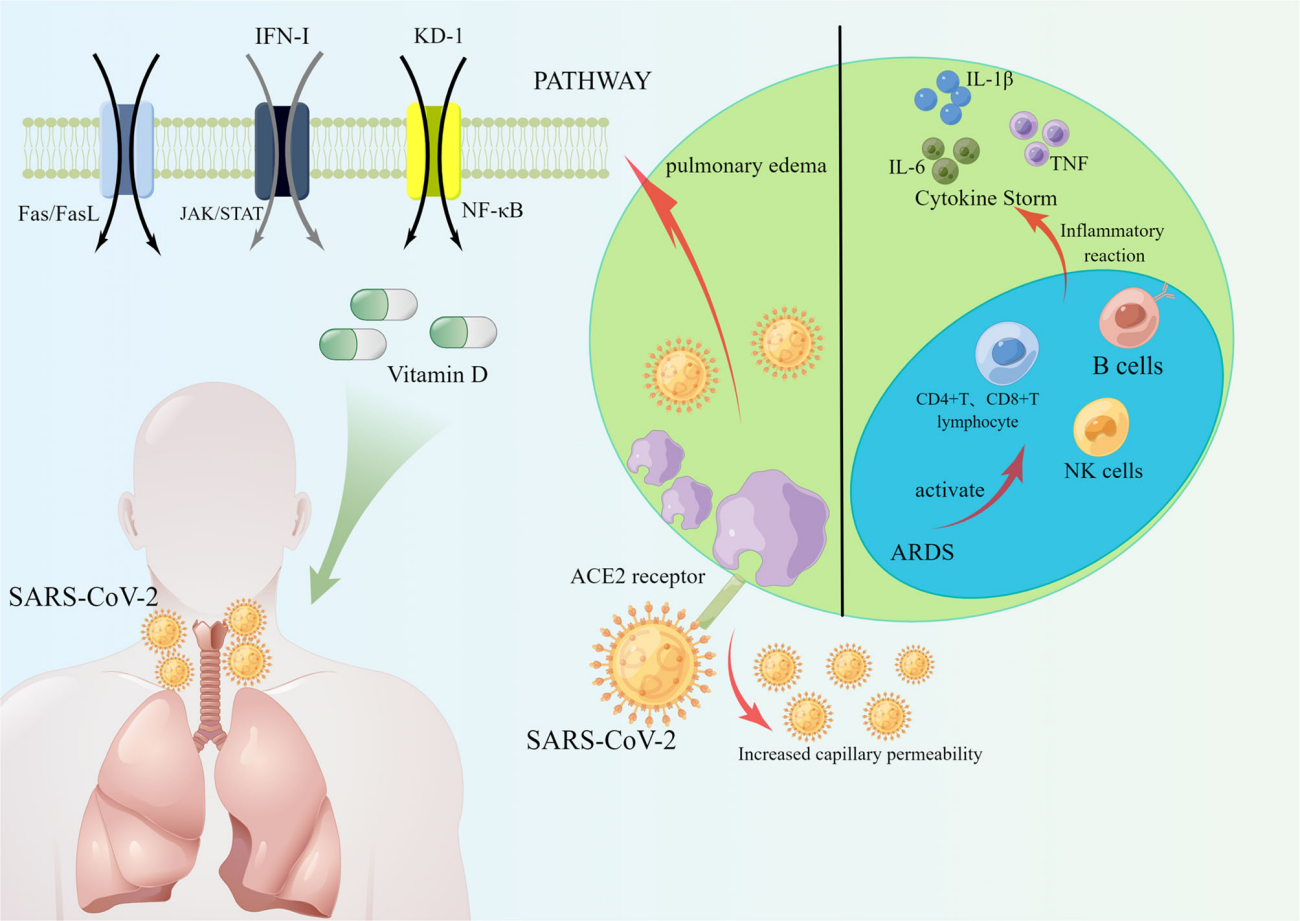
SARS-CoV-2 enters cells through ACE2 receptors, and ACE2 increases capillary permeability, allowing more SARS-CoV-2 to enter cells, which in turn induces the development of ARDS. COVID-19 activated an immune response in which NK cells, CD4+ T lymphocytes, CD8+ T lymphocytes and B cells reacted immunologically in COVID-19 patients, causing the production of cytokines IL-6, IL-1β, TNF, etc. Reducing COVID-19 infection and prevent ARDS through Fas/FasL pathway, JAK/STAT pathway, and NF-κB pathway. Helping patients to prevent and treat COVID-19 by increasing type I interferon and vitamin D.

SARS-CoV-2 S protein in the respiratory tract with respect to alveolar type 2 epithelial cells angiotensin-converting enzyme 2. ACE2 was found to be a major indicator of mortality in COVID-19 patients, and overexpression of ACE2 enhanced viral entry. ACE2 is a transmembrane type I glycoprotein with two functional domains, the N-terminal peptidase domain, and the C-terminal domain, whose physiological role is to control blood pressure and vasoconstriction. SARS-CoV-2 S protein binding to ACE2 protein invades alveolar epithelial cells (Seyed Hosseini et al., 2020), inducing ARDS and leading to death in most patients (Balkhi, 2021). This shows that maintaining normal ACE2 levels in the lung is crucial. The study (Datta et al., 2020) found that ACE2 was expressed at high levels in the epithelial cells of the lung after the death of COVID-19 patients and that targeting the ACE2/Ang 1-7 axis and blocking the S-protein interaction of ACE2 with SARS-CoV-2 to prevent the entry of SARS-CoV-2 into the cells could be used to treat and prevent COVID-19. The study demonstrated (van Eeden et al., 2020a) that ACE2 is the cellular receptor for SARS-CoV-2 entry into the host and that the high affinity of the receptor-binding domain (RBD) of the SARS-CoV-2 S protein for the ACE2 receptor accelerates the spread of SARS-CoV-2 (Chilamakuri and Agarwal, 2021) (**Figure 2**).

The study has pointed out the key role of ACE2 in acute lung injury through a clinical report of COVID-19 patients, in which ACE, angiotensin II, and angiotensin II type 1a receptor (AT1a) promote the development of the disease and induce pulmonary edema causing impairment of lung function (Verdecchia et al., 2020). It has been shown by mouse models that ACE-deficient mice have a significant improvement in lung function and pulmonary edema, and that recombinant ACE2 prevents mice from suffering acute lung injury. However, other contrary study found the mechanism of ACE2-causing lung injury in experiments with data from acute lung injury models as a decrease in intrapulmonary ACE2 levels, leading to increased vascular permeability, inflammatory cell aggregation, and severe hypoxia causing pulmonary edema (Imai et al., 2005). In conclusion, maintaining normal intrapulmonary ACE2 levels inhibits ARDS, while the mechanisms by which high or low ACE2 levels affect ARDS still need to be further explored and determined (**Figure 2**).

### 3.2 ARDS due to Cytokine Storm Triggered by SARS-CoV-2 Infection

Recently, a large number of domestic and international experts have pointed out that the severity of COVID-19 is associated with cytokine storm and that the overproduction of pro-inflammatory factors leads to acute respiratory distress syndrome and accelerates the death of patients. Cytokines are produced by a variety of immune cells, including natural killer cells and adaptive T and B lymphocytes (Ragab et al., 2020), which play an important regulatory role in the response to antiviral immunity and inflammation (Tisoncik et al., 2012). Cytokine storm (CS) refers to a series of clinical disorders caused by immune response disorders (Kim et al., 2021), such as inflammatory lung injury and ARDS (Li et al., 2020b), accompanied by the rapid production of large amounts of cytokines (IL-1β, IL-1RA, IL-7, IL-8, IL-10, IFN-γ, and TNF-α) (**Figure 2**).

It has been clinically found that serum pro-inflammatory factor levels are elevated in COVID-19 patients, and the severity of COVID-19 patients increases (Anka et al., 2021). Due to the abnormal release of pro-inflammatory factors that disrupt the pulmonary microvascular and alveolar epithelial cell barriers, leading to alveolar edema and hypoxia (Ragab et al., 2020; Balkhi, 2021). In clinical applications, it has been found that severe COVID-19 patients presenting with ARDS lead to an increase in pro-inflammatory factors, most notably interleukin 1β (IL-1β), interleukin-6 (IL-6), and TNF (Anka et al., 2021). In an analysis of cytokine-responsive gene sets, one author (Lee et al., 2020) found a significant TNF/IL-1β inflammatory response in lung tissue and that the severity of COVID-19 disease was accompanied by a TNF/IL-1β response.IL-6 is a family of cytokines involved in immune cell differentiation and activation (Sun et al., 2020), and the latest il-6 inhibitor pertuzumab has the potential to treat COVID-19 (Gu et al., 2020) (**Figure 2**).

### 3.3 SARS-CoV-2 Activates the Immune Response Leading to the ARDS

SARS-CoV-2 infection can activate both innate and adaptive immune responses, and SARS-CoV-2 may lead to too long a delay in the innate immune response as well as an inability to initiate an adaptive immune response for a long time, leading to severe lung disease (Sette and Crotty, 2021).

The innate immune sensing mechanism is the first line of defense against viruses and is an important aspect of viral immunity (Anka et al., 2021). SARS-CoV-2 innate immune responses are initially stimulated by pulmonary epithelial cells, alveolar macrophages, and neutrophils, which then trigger an adaptive immune response involving T and B lymphocytes (Toor et al., 2021). The innate immune response limits viral replication within infected cells, creating an antiviral state in the local tissue environment and initiating an adaptive immune response (Sette and Crotty, 2021). SARS-CoV-2 has four basic components of adaptive immunity: NK cells, B cells, CD4+ T cells, and CD8+ T cells. In COVID-19 patients, total lymphocytes, CD4+ T cells, CD8+ T cells, B cells, and NK cells were found to be decreased (Wang et al., 2020a; Anka et al., 2021), and for data from blood tests at admission, lymphocyte counts were found to be significantly lower in patients with severe disease than in patients with mild disease (0.9×10^9^ cells/L [range 0.8–0.9] versus 1.2 [1.0–1.6]; p = 0.011) (Zhou et al., 2020). The possible mechanisms of lung injury caused by these cells will be summarized below (**Figure 1**).

#### 3.3.1 Natural Killer Cells

Natural killer cells are required for the control of viral infections, thus restoring NK cell function has the potential to overcome the immune homeostasis required for COVID-19 infection. NK cells play a crucial role in regulating the immune response, not only by forming part of the innate immune system but also by regulating the adaptive immune response (Schuster et al., 2016; van Eeden et al., 2020b). Recent studies have highlighted that NK cells are regulatory cells that interact with dendritic cells, macrophages, T cells, and endothelial cells (Vivier et al., 2008).NK cells can limit or exacerbate the immune response. The role of NK cells as effectors against transformed and virally infected cells has been demonstrated (Schuster et al., 2016). SARS-CoV-2 infection in alveolar pneumocytes, resident alveolar macrophages, epithelial cells of the alveoli, and damaged lung cells trigger an inflammatory response with the release of cytokines and chemokines. This response further attracts natural killer cells, which enter the alveoli from the bloodstream and exacerbate the inflammatory response. The worsening of excessive inflammation triggers a cytokine storm that eventually progresses to ARDS and even death (Satarker et al., 2021). NKG2A is a receptor expressed on NK cells that induces IFN-γ and TNF-α (André et al., 2018; van Eeden et al., 2020b), and NKG2A expression by NK cells is activated in the peripheral and pulmonary microenvironment of patients with COVID-19 (Hammer et al., 2022). The number of NK cells is reduced in patients with COVID-19, and the function of NK cells is depleted as NKG2A expression increases in patients. Importantly, after treatment and recovery, the number of NK cells in these patients recovered with the decrease in NKG2A expression (Zheng et al., 2020). Chemokines MCP-1 and IP-10 recruit NK cells to inflammatory tissues, yet NK cell cytotoxicity and immune regulation are diminished, leading to an inflammatory response in SARS-CoV-2 infected patients (van Eeden et al., 2020b). This could suggest that the functional depletion of NK cells is associated with infection with SRAS-CoV-2 (**Figure 2**).

#### 3.3.2 CD4+T and CD8+T Lymphocytes

T lymphocytes, CD4+T and CD8+T, play a key role in autoimmune and inflammatory responses (Wang et al., 2020b). Some speculations have been made on the mechanism of lung injury caused by SARS-CoV-2 (Li et al., 2020a). SARS-CoV-2 may act mainly on T lymphocytes, which in turn leads to the deterioration of the patient’s condition. It has been shown that T cells undergo immune activation and antiviral immune responses triggered by SARS-CoV-2 and induce infected cell death (Toor et al., 2021). However, the pathogenic synergy between T cell-associated bystander effects and the more pronounced training innate immune effects in adults than in children can lead to abnormal or excessive immune responses in SARS-CoV-2 infected patients, which in turn can lead to tissue damage (Li et al., 2020a; de Candia et al., 2021). T lymphocyte subsets can be divided into CD4+ T cells and CD8+ T cells.19 CD4+T and CD8+ T cells in patients with nCoV are important in clearing infected cells by inducing immune damage SRAS-CoV-2 plays a very important role (Satarker et al., 2021). The study suggested (Zheng et al., 2020) that the functional failure of CD8+ T cells was associated with SRAS-CoV-2 infection. This shows the significance of CD8+ T cells for COVID-19. S protein is an important structural protein in SARS-CoV-2 that mediates the entry of SARS-CoV-2 into host cells (Chilamakuri and Agarwal, 2021). The importance of T lymphocytes in viral clearance and recovery is also greatly implied by the fact that CD4+ and CD8+ T cells in most recovered patients produce a large number of antiviral immune responses against S proteins (Toor et al., 2021). Another study (Grifoni et al., 2020)also identified circulating SARS-CoV-2 specific CD8+ T cells and CD4+ T cells in COVID-19 recovered patients and also found that CD4+ T cells were positively correlated with the size of anti-SARS-CoV-2 IgG and IgA titers, and anti-acute RBD antibody responses made by COVID-19 patients were positively correlated with the size of specific CD4+ T and CD8+ T cell responses (**Figure 2**).

#### 3.3.3 B Cells

B cells mainly perform humoral immunity of the body and can differentiate into plasma cells upon antigen stimulation, which can further synthesize specific antibodies and play a certain role in immune protection. In response to most viral infections, B cells can bind to viral proteins through their antigen receptors, by secreting effector molecules (IL-2, IL-4, IL-6, IFN-γ, TNF-α) to help contain viral infections (Rodda et al., 2021). Studies have shown that the serum-neutralizing antibody response to SARS-CoV-2 spiking proteins occurs within two weeks after the onset of symptoms, but memory B cells can be rapidly reactivated after secondary infection to help prevent SARS-CoV-2 infection and death (Lee and Oh, 2021). The mechanism of action lies in the fact that B cells can differentiate into plasma cells or form germinal centers through extrafollicular antibody (EF) responses, and in COVID-19 patients, antibodies produced by EF responses effectively neutralize SARS-CoV-2 (Luo and Yin, 2021). Patients recovering from COVID-19 produce specific immunoglobulin (IgG) antibodies that neutralize plasma and memory B cells that persist for at least 3 months. SARS-CoV-2-specific IgG memory B cells increase over time and memory B cell receptors neutralize the virus. Thus COVID-19 patients produce memory cells and display antiviral immunity (Luo and Yin, 2021) (**Figure 2**).

### 3.4 COVID-19 Triggers Fas/FasL Signaling Pathway Pro-Apoptosis Causing the ARDS

Fas is a tumor necrosis cell surface receptor factor, which has long been considered a death receptor and maintains immune homeostasis by mediating cell apoptosis (Bellesi et al., 2020). The pro-apoptotic role of the Fas/FasL signaling pathway is important in the development of acute lung injury (ALI). The Fas pathway has been studied as a potential contributor to lung inflammation and alveolar epithelial cell apoptosis in patients with ALI. The Fas pathway is activated by activation by FasL binding to Fas on the cell surface, leading to an intracellular cascade response that results in inflammation and apoptosis of Fas-bearing cells. It has been found (Andre et al., 2022) that activated T cells are susceptible to death *via* the Fas/FasL signaling pathway and that sFasL and Fas/CD95 expression correlated positively with T cell apoptosis in the plasma of COVID-19 patients. Selective blockade of Fas was found to attenuate lung injury in animal models, and lung injury could result from Fas activation (Glavan et al., 2011). Another study found that SARS-CoV-2 entered the airway and infected mainly fine bronchial epithelial cells and alveolar epithelial cells, causing local inflammation associated with lung injury (Rendeiro et al., 2021). In contrast, Fas/FasL, an apoptotic mediator, can induce inflammation by releasing pro-inflammatory factors that cause the migration of neutrophils and macrophages to the site of injury (Yu and Fehlings, 2011), and the Fas death receptor is normally expressed on lung epithelial cells. It is hypothesized that COVID-19 infection acts on alveolar epithelial cells to activate the Fas/FasL signaling pathway, which in turn induces abnormal apoptosis and causes acute lung injury (**Figures 1** and **2**).

### 3.5 COVID-19 Triggers Inflammation Through the JAK/STAT Pathway Causing the ARDS

COVID-19 virus induces activators of message transcription and transcription 1 (STAT1) dysfunction and compensatory hyperactivation of STAT3. The recruitment and subsequent activation of innate immune cells in the infected lung drive the destruction of lung structures, which leads to regional endothelial cell infection and the formation of a hypoxic environment where over-produced PAI-11 binds to TLR4 on macrophages and induces the secretion of pro-inflammatory cytokines and chemokines (Matsuyama et al., 2020). The JAK-STAT signaling pathway refers to the phosphorylation and dimerization of STAT by JAK, followed by its passage through the nuclear membrane translocation to the nucleus to regulate the expression of related genes, which in turn causes a cytokine storm (Zhang et al., 2020a). Some investigators have found in the pathology of SARS-CoV-2 disease that concentrations of pro-inflammatory cytokines and chemokines correlate with disease severity and adverse clinical outcomes, and that levels of pro-inflammatory factors (IL-2, IL-4, IL-6, IL-7, IL-10, TNF-α, and IFN-γ) and chemokines (CCL2, CCL8) are significantly elevated, IL-6 has been shown to activate the JAK-STAT signaling pathway, giving the body an immunomodulatory function, and thus it is hypothesized that the JAK-STAT signaling pathway is a key part of immunity and immunopathology during COVID-19 infection (Luo et al., 2020). The SARS-CoV-2 infection triggers inflammation through the JAK/STAT pathway, leading to lung cells, endothelial cells, macrophages, monocytes, lymphocytes, natural killer cells, and dendritic cells recruitment towards cytokine storm and eventual development of ARDS or even death (Satarker et al., 2021). Today an increasing number of anti-inflammatory drugs are used in the treatment of COVID-19, among which JAK inhibitors, are predicted to be of particular importance in the treatment of SARS-CoV-2 disease (Zhang et al., 2020b) (**Figures 1** and **2**).

### 3.6 COVID-19 Triggers Inflammation *via* NF-κB Pathway Causing the ARDS

The severity of COVID-19 is related to the activation of the host immune response, especially the inflammatory response, and an excessive inflammatory response will trigger lung injury. Therefore, limiting the dysfunctional inflammatory response is a critical step in anti-SARS-CoV-2 therapy. It was found (Wu et al., 2021) that SARS-CoV-2 infection leads to the development of an inflammatory response and the release of multiple cytokines by promoting the activation of the NF-κB signaling pathway. The mechanism was experimentally found to be that the nucleocapsid (N) protein in SARS-CoV-2, after binding to viral RNA, undergoes liquid-liquid phase separation (LLPS) to recruit TAK1 and IKK complexes, thus promoting the activation of NF-κB signaling to enhance NF-κB activation. Not only that, but LLPS inhibitors can also attenuate the phase separation of N proteins and limit their regulatory function in NF-κB activation. It has been shown that SARS-CoV-2 ORF3a, M, ORF7a and N proteins are NF-κB activators and that activation is positively correlated with an increase in the amount of viral protein, indicating a dose-dependent activation of NF-κB by SARS-CoV-2 ORF3A, M, ORF7a, and N proteins.SARS-CoV-2 ORF7a protein is the most potent inducer of NF-KB, activating NF-κB signaling and promoting the production of pro-inflammatory cytokines (Su et al., 2021). Recent studies have found that a variety of herbs can inhibit SARS-CoV-2 infection through the NF-κB signaling pathway. Liushen capsule (LS), a traditional Chinese medicine, has anti-inflammatory, antiviral, and immunomodulatory activity properties. Some scholars (Ma et al., 2020) detected the expression of key proteins in the NF-κB/MAPK signaling pathway by protein blotting and found that LS could inhibit SARS-CoV-2 virus infection by downregulating the expression of inflammatory cytokines-induced viruses and regulating the activity of NF-κB/MAPK signaling pathway *in vitro* (**Figure 2**).

Alveolar hypercoagulation and fibrinolytic inhibition are important features in ARDS, and they are closely associated with severe hypoxemia, which is one of the important reasons why ARDS is difficult to cure. Several studies have now found that the NF-KB pathway can be involved in regulating endotoxin (LPS)-induced alveolar hypercoagulation and fibrinolysis inhibition. By targeting the NF-KB signaling pathway, alveolar hypercoagulation and fibrinolytic inflammation in ARDS could be improved. It is further hypothesized that the NF-KB signaling pathway can be targeted to reduce the occurrence of inflammatory response and cytokine release, inhibit SARS-CoV-2 infection, and improve alveolar hypercoagulation and fibrinolytic inhibition in ARDS (Pooladanda et al., 2019; Yang et al., 2021) (**Figure 1**).

### 3.7 COVID-19 Inhibits IFN-Is Causing the ARDS

Type I interferon (IFN-I) was first discovered in the classical experiments of Isaacs and Lindenman, and IFN-Is were found to have antiviral activity (Schreiber, 2020). The association of IFN-I with lung epithelial barrier function has been demonstrated and some researchers have found that the protective effect of IFN-I can be used in acute lung injury, where the loss of IFN-I signal leads to a significant reduction in barrier function caused by alveolar epithelial type II cell death (Maier et al., 2016). Type I interferons (IFN-Is) secreted by cells are rapidly produced upon viral infection and exhibit their antiviral activity in controlling virus proliferation and dissemination as well as effective antiviral immune responses (King and Sprent, 2021), which, if the immune response is unbalanced, leads to diminished production of IFN-Is and accelerated release of pro-inflammatory cytokines, and eventually, the disease becomes severe (Sa Ribero et al., 2020).SARS-CoV-2 has evolved mechanisms to inhibit IFN-I production to evade the inhibitory effects of IFN-I, by impairing the adaptive immune response and exacerbating inflammatory disease in the late stages of viral infection, ultimately affecting the course and efficiency of disease transmission (King and Sprent, 2021). Currently, in SARS-CoV-2 pneumonia, SARS-CoV-2 is found to cause a delay in IFN-I-mediated defense against viral functions and a large production of cytokines (IFN-α, IFN-β, IL-1, IL-6) (Nilsson-Payant et al., 2021), ultimately maintaining a balance of deleterious host responses.SARS-CoV-2 infection inhibits IFN-I production, impairs the adaptive immune response, and exacerbates inflammatory disease in the late stages of infection. Therefore, clarifying the etiology of SARS-CoV-2 disease is important to investigate the defective responsiveness of IFN-I (King and Sprent, 2021). Hoagland et al. (2021) found reduced viral load and tissue damage by intranasal injection of IFN-I, which reflects that IFN-I can limit viral replication and inflammation. Systemic administration of interferon can lead to some unwanted side effects, but local injection of IFN-I through the respiratory tract has shown excellent recovery in patients with COVID-19, further suggesting that this method may become an effective early intervention in respiratory disease caused by SARS-CoV-2 (**Figures 1** and **2**).

### 3.8 COVID-19 Causes ARDS Through the Vitamin D Pathway

Vitamin D maintains the balance of blood calcium and blood phosphorus, and for those who are deficient in vitamin D, proper supplementation can enhance our immune system and will bring some help in fighting SARS-CoV-2. Many studies today show that COVID-19 patients who are vitamin D deficient usually have a poor prognosis, while patients with high levels of vitamin D have an even better prognosis. Vitamin D is an immunomodulatory hormone whose active form, 1,25 dihydroxy vitamin D (1,25(OH)D), binds to the vitamin D receptor (VDR) to exert anti-inflammatory and immunomodulatory effects, prevent inflammatory response, and accelerate the healing process in affected areas, and it acts mainly in lung tissue with proven effectiveness against various upper respiratory tract infections (Mohan et al., 2020). One study (Rhodes et al., 2021) found that vitamin D deficiency is also related to the severity of respiratory diseases in children. Vitamin D has also been found by many scientists to affect ACE2 by increasing the ratio of ACE2 to ACE, thereby increasing angiotensin II hydrolysis and decreasing the subsequent inflammatory cytokine response to pathogens and lung injury (Mohan et al., 2020; Xiao et al., 2021). Since ACE2 is the host cell receptor for SARS-CoV-2, vitamin D could attenuate acute lung injury and ARDS by affecting ACE2 (Xiao et al., 2021). However, Ghasemian et al (Ghasemian et al., 2021), focused on the role of vitamin D in patients with COVID-19 and entered a meta-analysis of 23 studies in 11,901 subjects, which found that vitamin D deficiency had no substantial effect on mortality in patients with COVID-19. However, patients with low vitamin D levels or vitamin D deficiency had a higher risk of developing severe disease (**Figures 1** and **2**).

## 4 Conclusions and Challenges

COVID-19 is a fatal respiratory infectious disease caused by SARS-CoV-2, which can cause the ARDS in severe cases, resulting in lung injury and accelerating the death of patients. In order to reduce the mortality of COVID-19 and gain insight into the study of SARS-CoV-2 causing the ARDS and to develop a broad spectrum of drugs as early as possible, this paper analyzes the possible mechanisms of SARS-CoV-2 causing ARDS in angiotensin-converting enzyme 2, cytokine storm, immune cells, Fas/FasL pathway, JAK/STAT pathway, NF-κB pathway, type I interferon, and vitamin D. Their importance in COVID-19 and the possible mechanisms of lung damage are described. In the future, there are still some areas that need attention and research in the area of SARS-CoV-2 causing the acute respiratory distress syndrome (**Figure 1**).

In terms of ACE2, ACE2 receptor is the key entry point for viral to entry cells, and genetic mutations in ACE2 may affect expression levels as well as protein conformation and stability, which may change the affinity of the SARS-CoV-2 S protein, making individuals more resistant or susceptible to viral infection (Antony and Vijayan, 2021). This latest speculation provides a future understanding of the clinically relevant COVID-19 pathophysiological response brings significant help for the future understanding of COVID-19 clinically relevant pathophysiological response and provides ideas for vaccine development. In terms of cytokine storm, the cytokine storm triggered by COVID-19 exacerbates disease progression and leads to immune disorders, which provides a potential approach to treat COVID-19 by early identification of cytokine storm and anti-inflammatory therapy that reduces cytokine response, ultimately leading to reduced morbidity and mortality. There are a number of anti-cytokine approaches that have been shown to be effective in the treatment of cytokine storm syndrome, such as anti-IL-1, IL-6, and IFN-γ drugs (Nile et al., 2020), but further experiments are needed to determine which drugs are effective in patients with cytokine storm syndrome caused by SARS-CoV-2. In terms of immune response, NK cells control cellular infection and play a key role in maintaining immune homeostasis, and SARS-CoV-2 infection controls NK cell function, thereby disrupting this balance. B cells also play a very important role in maintaining humoral immunity in the body, and the persistence of the immune response is essential to prevent reinfection in patients recovering from COVID-19, and persistent humoral Immunity is mediated by memory B cells and memory B cells can provide protection against re-infection of the body with viruses (Ogega et al., 2021), but there are still many gaps in knowledge and understanding of the immune memory response to SARS-CoV-2. It is hoped that future drug development will be carried out from the perspective of improving NK cell function and exploiting the memory response of B cells. It has been shown that physical inactivity decreases NK cell activity and IFN-γ expression (van Eeden et al., 2020b). This suggests the importance of increased exercise in patients with COVID-19. CD4+ T cells as coordinators of antiviral immune responses can enhance the effector function of CD8+ T cells or directly kill infected cells (Meckiff et al., 2020). CD4+ T cells and CD8+ T cells-mediated immune responses are present throughout the course of COVID-19 disease, but evidence of CD4+ T cells and CD8+ T cells in COVID-19 disease surveillance is still lacking in large samples. It is hoped that the dynamic detection of CD4+ T and CD8+ T cells can be used in the clinic in the future to grasp the patient’s disease and eventually eliminate SARS-CoV-2 and prevent the patient from progressing to ARDS (**Figure 2**).

Fas maintains immune homeostasis and the Fas/FasL pathway mediates the formation of ALI. sFasL, Fas/CD95 expression was also found in COVID-19 patients (Glavan et al., 2011). It is hypothesized that COVID-19 infection activates the Fas/FasL signaling pathway, induces abnormal apoptosis, and causes ARDS. COVID-19 infection triggers inflammation through the JAK/STAT pathway, causing a cytokine storm that eventually develops into ARDS. The pro-inflammatory factor IL-6 has been shown to activate the JAK/STAT signaling pathway (Luo et al., 2020), and more experiments are needed to prove whether other pro-inflammatory factors in COVID-19 patients have the same effect. SARS-CoV-2 ORF3a, M, ORF7a and N proteins can promote activation of the NF-κB signaling pathway, with ORF7a being the most potent activator leading to the development of inflammatory responses and cytokine release (Su et al., 2021). the NF-κB pathway can be involved in the regulation of LPS and ameliorate ARDS-induced alveolar hypercoagulation and fibrinolysis inhibition. Although the viral proteins that activate the inflammatory response and their molecular mechanisms are not yet known. However, it can be speculated that by targeting the NF-κB signaling pathway, SARS-CoV-2 infection and inflammatory response can be effectively inhibited and the symptoms of ARDS can be improved. In summary, by inhibiting several aspects of the Fas/FasL signaling pathway, NF-κB signaling pathway, and JAK/STAT pathway, all of them can reduce the infection of COVID-19 to some extent and further prevent the development of ARDS. Future studies can focus on these three pathways as well as other pathways to provide new strategies for the treatment of COVID-19. Increasing type I interferon and vitamin D has a good effect on the prevention and treatment of COVID-19. In the future, intranasal intake of IFN-I may be used as an early treatment to eliminate the virus. For people who lack vitamin D, appropriate vitamin D supplementation can enhance the immune ability and bring some help to fight the virus However, it is still unclear to what extent vitamin D helps patients with COVID-19, and there is a lack of sufficient favorable evidence. (**Figure 2**).

Currently, ARDS remains a state of high morbidity and mortality due to the lack of specific drugs, supportive treatment by mechanical ventilation and non-mechanical ventilation, and long-term sequelae of patients after treatment. Therefore, research on the molecular and physiological mechanisms of ARDS needs to be further improved to develop specific drugs and find better treatment strategies to reduce the mortality caused by this syndrome.

Nowadays, people all over the world are engaged in the fight against SARS-CoV-2, and the mechanisms of SARS-CoV-2-induced acute respiratory distress syndrome are playing an increasingly important role in understanding the virus, fighting the epidemic, and developing drugs. It is expected that in the future, we will have a deeper understanding of the SARS-CoV-2 and be able to supplement the understanding of the mechanism of acute respiratory distress syndrome with more experimental data and clinical observations, to contribute to the prevention and control of the epidemic as well as to the medical field.

## Author Contributions

JZ and RGuo: writing and visualization. JM, ZF, and JG: reviewing and editing. XK, RGao, and LY: conceptualization and supervision. All authors contributed to the article and approved the submitted version.

## Funding

This study was supported by the Scientific research project of Tianjin Education Commission (Grant No. 2021KJ134 to XK); Tianjin Municipal Education Commission Scientific Research Project (Natural Science, Grant No. 2019ZD11 to LY); Scientific and technological innovation project of China Academy of Chinese Medical Sciences (Grant No. C12021A04701 to RG).

## Conflict of Interest

The authors declare that the research was conducted in the absence of any commercial or financial relationships that could be construed as a potential conflict of interest.

## Publisher’s Note

All claims expressed in this article are solely those of the authors and do not necessarily represent those of their affiliated organizations, or those of the publisher, the editors and the reviewers. Any product that may be evaluated in this article, or claim that may be made by its manufacturer, is not guaranteed or endorsed by the publisher.

## References

[B1] André P. Denis C. Soulas C. Bourbon-Caillet C. Lopez J. Arnoux T.. (2018). Anti-NKG2A mAb Is a Checkpoint Inhibitor That Promotes Anti-Tumor Immunity by Unleashing Both T and NK Cells. Cell 175, 1731–1743.e13. doi: 10.1016/j.cell.2018.10.014 30503213 PMC6292840

[B2] Andre S. Picard M. Cezar R. Roux-Dalvai F. Alleaume-Butaux A. Soundaramourty C.. (2022). T Cell Apoptosis Characterizes Severe Covid-19 Disease. Cell Death Differ 22, 1–14. doi: 10.1038/s41418-022-00936-x PMC8782710 35066575

[B3] Anka A. U. Tahir M. I. Abubakar S. D. Alsabbagh M. Zian Z. Hamedifar H.. (2021). Coronavirus Disease 2019 (COVID-19): An Overview of the Immunopathology, Serological Diagnosis and Management. Scand. J. Immunol. 93, e12998. doi: 10.1111/sji.12998 33190302 PMC7744910

[B4] Antony P. Vijayan R. (2021). Role of SARS-CoV-2 and ACE2 Variations in COVID-19. BioMed. J. 44, 235–244. doi: 10.1016/j.bj.2021.04.006 34193390 PMC8059258

[B5] Attaway A. H. Scheraga R. G. Bhimraj A. Biehl M. Hatipoğlu U. (2021). Severe Covid-19 Pneumonia: Pathogenesis and Clinical Management. BMJ (Clinical Res. ed.) 372, n436. doi: 10.1136/bmj.n436 33692022

[B6] Balkhi M. Y. (2021). Mechanistic Understanding of Innate and Adaptive Immune Responses in SARS-CoV-2 Infection. Mol. Immunol. 135, 268–275. doi: 10.1016/j.molimm.2021.04.021 33940513 PMC8084627

[B7] Batah S. S. Fabro A. T. (2021). Pulmonary Pathology of ARDS in COVID-19: A Pathological Review for Clinicians. Respir. Med. 176, 106239. doi: 10.1016/j.rmed.2020.106239 33246294 PMC7674971

[B8] Bellesi S. Metafuni E. Hohaus S. Maiolo E. Marchionni F. D'innocenzo S.. (2020). Increased CD95 (Fas) and PD-1 Expression in Peripheral Blood T Lymphocytes in COVID-19 Patients. Br. J. Haematol. 191, 207–211. doi: 10.1111/bjh.17034 32679621 PMC7405050

[B9] Chilamakuri R. Agarwal S. (2021). COVID-19: Characteristics and Therapeutics. Cells 10, 206. doi: 10.3390/cells10020206 33494237 PMC7909801

[B10] Datta P. K. Liu F. Fischer T. Rappaport J. Qin X. (2020). SARS-CoV-2 Pandemic and Research Gaps: Understanding SARS-CoV-2 Interaction With the ACE2 Receptor and Implications for Therapy. Theranostics 10, 7448–7464. doi: 10.7150/thno.48076 32642005 PMC7330865

[B11] De Candia P. Prattichizzo F. Garavelli S. Matarese G. (2021). T Cells: Warriors of SARS-CoV-2 Infection. Trends Immunol. 42, 18–30. doi: 10.1016/j.it.2020.11.002 33277181 PMC7664351

[B12] Ghasemian R. Shamshirian A. Heydari K. Malekan M. Alizadeh-Navaei R. Ebrahimzadeh M. A.. (2021). The Role of Vitamin D in the Age of COVID-19: A Systematic Review and Meta-Analysis. Int. J. Clin. Pract. 75, e14675. doi: 10.1111/ijcp.14675 34322971 PMC8420549

[B13] Gibson P. G. Qin L. Puah S. H. (2020). COVID-19 Acute Respiratory Distress Syndrome (ARDS): Clinical Features and Differences From Typical Pre-COVID-19 ARDS. Med. J. Aust. 213, 54–56.e1. doi: 10.5694/mja2.50674 32572965 PMC7361309

[B14] Glavan B. J. Holden T. D. Goss C. H. Black R. A. Neff M. J. Nathens A. B.. (2011). Genetic Variation in the FAS Gene and Associations With Acute Lung Injury. Am. J. Respir. Crit. Care Med. 183, 356–363. doi: 10.1164/rccm.201003-0351OC 20813889 PMC3056231

[B15] Grasselli G. Cattaneo E. Scaravilli V. (2021). Ventilation of Coronavirus Disease 2019 Patients. Curr. Opin. In Crit. Care 27, 6–12. doi: 10.1097/MCC.0000000000000793 33315636

[B16] Grifoni A. Weiskopf D. Ramirez S. I. Mateus J. Dan J. M. Moderbacher C. R.. (2020). Targets of T Cell Responses to SARS-CoV-2 Coronavirus in Humans With COVID-19 Disease and Unexposed Individuals. Cell 181, 1489–1501, e1415. doi: 10.1016/j.cell.2020.05.015 32473127 PMC7237901

[B17] Gu T. Zhao S. Jin G. Song M. Zhi Y. Zhao R.. (2020). Cytokine Signature Induced by SARS-CoV-2 Spike Protein in a Mouse Model. Front. In Immunol. 11, 621441. doi: 10.3389/fimmu.2020.621441 33584719 PMC7876321

[B18] Hammer Q. Dunst J. Christ W. Picarazzi F. Wendorff M. Momayyezi P.. (2022). SARS-CoV-2 Nsp13 Encodes for an HLA-E-Stabilizing Peptide That Abrogates Inhibition of NKG2A-Expressing NK Cells. Cell Rep. 38, 110503. doi: 10.1016/j.celrep.2022.110503 35235832 PMC8858686

[B19] Hoagland D. A. Moller R. Uhl S. A. Oishi K. Frere J. Golynker I.. (2021). Leveraging the Antiviral Type I Interferon System as a First Line of Defense Against SARS-CoV-2 Pathogenicity. Immunity 54 557–54570.e555. doi: 10.1016/j.immuni.2021.01.017 33577760 PMC7846242

[B20] Imai Y. Kuba K. Rao S. Huan Y. Guo F. Guan B.. (2005). Angiotensin-Converting Enzyme 2 Protects From Severe Acute Lung Failure. Nature 436, 112–116. doi: 10.1038/nature03712 16001071 PMC7094998

[B21] Khamis F. Memish Z. Bahrani M. A. Dowaiki S. A. Pandak N. Bolushi Z. A.. (2021). Prevalence and Predictors of in-Hospital Mortality of Patients Hospitalized With COVID-19 Infection. J. Infect. Public Health 14, 759–765. doi: 10.1016/j.jiph.2021.03.016 34022734 PMC8053361

[B22] Kim J. S. Lee J. Y. Yang J. W. Lee K. H. Effenberger M. Szpirt W.. (2021). Immunopathogenesis and Treatment of Cytokine Storm in COVID-19. Theranostics 11, 316–329. doi: 10.7150/thno.49713 33391477 PMC7681075

[B23] King C. Sprent J. (2021). Dual Nature of Type I Interferons in SARS-CoV-2-Induced Inflammation. Trends Immunol. 42, 312–322. doi: 10.1016/j.it.2021.02.003 33622601 PMC7879020

[B24] Lee E. Oh J. E. (2021). Humoral Immunity Against SARS-CoV-2 and the Impact on COVID-19 Pathogenesis. Mol. Cells 44, 392–400. doi: 10.14348/molcells.2021.0075 34059562 PMC8245316

[B25] Lee J. S. Park S. Jeong H. W. Ahn J. Y. Choi S. J. Lee H.. (2020). Immunophenotyping of COVID-19 and Influenza Highlights the Role of Type I Interferons in Development of Severe COVID-19. Sci. Immunol. 5, eabd1554. doi: 10.1126/sciimmunol.abd155 32651212 PMC7402635

[B26] Li D. Chen Y. Liu H. Jia Y. Li F. Wang W.. (2020a). Immune Dysfunction Leads to Mortality and Organ Injury in Patients With COVID-19 in China: Insights From ERS-COVID-19 Study. Signal Transduct Target Ther. 5, 62. doi: 10.1038/s41392-020-0163-5 32371949 PMC7198844

[B27] Li M. Guo W. Dong Y. Wang X. Dai D. Liu X.. (2020b). Elevated Exhaustion Levels of NK and CD8(+) T Cells as Indicators for Progression and Prognosis of COVID-19 Disease. Front. Immunol. 11, 580237. doi: 10.3389/fimmu.2020.580237 33154753 PMC7591707

[B28] Luo W. Li Y. X. Jiang L. J. Chen Q. Wang T. Ye D. W. (2020). Targeting JAK-STAT Signaling to Control Cytokine Release Syndrome in COVID-19. Trends Pharmacol. Sci. 41, 531–543. doi: 10.1016/j.tips.2020.06.007 32580895 PMC7298494

[B29] Luo W. Yin Q. (2021). B Cell Response to Vaccination. Immunol. Invest. 50, 780–801. doi: 10.1080/08820139.2021.1903033 33779464

[B30] Maier B. B. Hladik A. Lakovits K. Korosec A. Martins R. Kral J. B.. (2016). Type I Interferon Promotes Alveolar Epithelial Type II Cell Survival During Pulmonary Streptococcus Pneumoniae Infection and Sterile Lung Injury in Mice. Eur. J. Immunol. 46, 2175–2186. doi: 10.1002/eji.201546201 27312374 PMC5370074

[B31] Ma Q. Pan W. Li R. Liu B. Li C. Xie Y.. (2020). Liu Shen Capsule Shows Antiviral and Anti-Inflammatory Abilities Against Novel Coronavirus SARS-CoV-2 *via* Suppression of NF-kappaB Signaling Pathway. Pharmacol. Res. 158, 104850. doi: 10.1016/j.phrs.2020.104850 32360580 PMC7192119

[B32] Matsuyama T. Kubli S. P. Yoshinaga S. K. Pfeffer K. Mak T. W. (2020). An Aberrant STAT Pathway Is Central to COVID-19. Cell Death Differ 27, 3209–3225. doi: 10.1038/s41418-020-00633-7 33037393 PMC7545020

[B33] Meckiff B. J. Ramirez-Suastegui C. Fajardo V. Chee S. J. Kusnadi A. Simon H.. (2020). Imbalance of Regulatory and Cytotoxic SARS-CoV-2-Reactive CD4(+) T Cells in COVID-19. Cell 183, 1340–1353.e1316. doi: 10.1016/j.cell.2020.10.001 33096020 PMC7534589

[B34] Meyer N. J. Gattinoni L. Calfee C. S. (2021). Acute Respiratory Distress Syndrome. Lancet 398, 622–637. doi: 10.1016/S0140-6736(21)00439-6 34217425 PMC8248927

[B35] Mohan M. Cherian J. J. Sharma A. (2020). Exploring Links Between Vitamin D Deficiency and COVID-19. PLoS Pathog. 16, e1008874. doi: 10.1371/journal.ppat.1008874 32946517 PMC7500624

[B36] Nile S. H. Nile A. Qiu J. Li L. Jia X. Kai G. (2020). COVID-19: Pathogenesis, Cytokine Storm and Therapeutic Potential of Interferons. Cytokine Growth Factor Rev. 53, 66–70. doi: 10.1016/j.cytogfr.2020.05.002 32418715 PMC7204669

[B37] Nilsson-Payant B. E. Uhl S. Grimont A. Doane A. S. Cohen P. Patel R. S.. (2021). The NF-κb Transcriptional Footprint Is Essential for SARS-CoV-2 Replication. J. Virol. 95, e0125721. doi: 10.1128/JVI.01257-21 34523966 PMC8577386

[B38] Ntatsoulis K. Karampitsakos T. Tsitoura E. Stylianaki E.-A. Matralis A. N. Tzouvelekis A.. (2021). Pulmonary Fibrosis and COVID-19: The Potential of Autotaxin as a Therapeutic Target. Front. In Immunol. 12, 687397. doi: 10.3389/fimmu.2021.687397 34671341 PMC8522582

[B39] Ogega C. O. Skinner N. E. Blair P. W. Park H. S. Littlefield K. Ganesan A.. (2021). Durable SARS-CoV-2 B Cell Immunity After Mild or Severe Disease. J. Clin. Invest. 131, e145516. doi: 10.1172/JCI145516 PMC8011891 33571162

[B40] Pooladanda V. Thatikonda S. Bale S. Pattnaik B. Sigalapalli D. K. Bathini N. B.. (2019). Nimbolide Protects Against Endotoxin-Induced Acute Respiratory Distress Syndrome by Inhibiting TNF-Alpha Mediated NF-kappaB and HDAC-3 Nuclear Translocation. Cell Death Dis. 10, 81. doi: 10.1038/s41419-018-1247-9 30692512 PMC6349848

[B41] Quesada-Gomez J. M. Entrenas-Castillo M. Bouillon R. (2020). Vitamin D Receptor Stimulation to Reduce Acute Respiratory Distress Syndrome (ARDS) in Patients With Coronavirus SARS-CoV-2 Infections: Revised Ms SBMB 2020_166. J. Steroid Biochem. Mol. Biol. 202, 105719. doi: 10.1016/j.jsbmb.2020.105719 32535032 PMC7289092

[B42] Ragab D. Salah Eldin H. Taeimah M. Khattab R. Salem R. (2020). The COVID-19 Cytokine Storm; What We Know So Far. Front. In Immunol. 11, 1446. doi: 10.3389/fimmu.2020.01446 32612617 PMC7308649

[B43] Rendeiro A. F. Ravichandran H. Bram Y. Chandar V. Kim J. Meydan C.. (2021). The Spatial Landscape of Lung Pathology During COVID-19 Progression. Nature 593, 564–569. doi: 10.1038/s41586-021-03475-6 33780969 PMC8204801

[B44] Rhodes J. M. Subramanian S. Laird E. Griffin G. Kenny R. A. (2021). Perspective: Vitamin D Deficiency and COVID-19 Severity - Plausibly Linked by Latitude, Ethnicity, Impacts on Cytokines, ACE2 and Thrombosis. J. Internal Med. 289, 97–115. doi: 10.1111/joim.13149 32613681 PMC7361294

[B45] Rodda L. B. Netland J. Shehata L. Pruner K. B. Morawski P. A. Thouvenel C. D.. (2021). Functional SARS-CoV-2-Specific Immune Memory Persists After Mild COVID-19. Cell 184, 169–183.e117. doi: 10.1016/j.cell.2020.11.029 33296701 PMC7682481

[B46] Sa Ribero M. Jouvenet N. Dreux M. Nisole S. (2020). Interplay Between SARS-CoV-2 and the Type I Interferon Response. PLoS Pathog. 16, e1008737. doi: 10.1371/journal.ppat.1008737 32726355 PMC7390284

[B47] Satarker S. Tom A. A. Shaji R. A. Alosious A. Luvis M. Nampoothiri M. (2021). JAK-STAT Pathway Inhibition and Their Implications in COVID-19 Therapy. Postgrad Med. 133, 489–507. doi: 10.1080/00325481.2020.1855921 33245005 PMC7784782

[B48] Schreiber G. (2020). The Role of Type I Interferons in the Pathogenesis and Treatment of COVID-19. Front. Immunol. 11, 595739. doi: 10.3389/fimmu.2020.595739 33117408 PMC7561359

[B49] Schuster I. S. Coudert J. D. Andoniou C. E. Degli-Esposti M. A. (2016). "Natural Regulators": NK Cells as Modulators of T Cell Immunity. Front. Immunol. 7, 235. doi: 10.3389/fimmu.2016.00235 27379097 PMC4905977

[B50] Sette A. Crotty S. (2021). Adaptive Immunity to SARS-CoV-2 and COVID-19. Cell 184, 861–880. doi: 10.1016/j.cell.2021.01.007 33497610 PMC7803150

[B51] Seyed Hosseini E. Riahi Kashani N. Nikzad H. Azadbakht J. Hassani Bafrani H. Haddad Kashani H. (2020). The Novel Coronavirus Disease-2019 (COVID-19): Mechanism of Action, Detection and Recent Therapeutic Strategies. Virology 551, 1–9. doi: 10.1016/j.virol.2020.08.011 33010669 PMC7513802

[B52] Sun X. Wang T. Cai D. Hu Z. Chen J. Liao H.. (2020). Cytokine Storm Intervention in the Early Stages of COVID-19 Pneumonia. Cytokine Growth Factor Rev. 53, 38–42. doi: 10.1016/j.cytogfr.2020.04.002 32360420 PMC7182527

[B53] Su C. M. Wang L. Yoo D. (2021). Activation of NF-kappaB and Induction of Proinflammatory Cytokine Expressions Mediated by ORF7a Protein of SARS-CoV-2. Sci. Rep. 11, 13464. doi: 10.1038/s41598-021-92941-2 34188167 PMC8242070

[B54] Tisoncik J. R. Korth M. J. Simmons C. P. Farrar J. Martin T. R. Katze M. G. (2012). Into the Eye of the Cytokine Storm. Microbiol. Mol. Biol. Rev. MMBR 76, 16–32. doi: 10.1128/MMBR.05015-11 22390970 PMC3294426

[B55] Toor S. M. Saleh R. Sasidharan Nair V. Taha R. Z. Elkord E. (2021). T-Cell Responses and Therapies Against SARS-CoV-2 Infection. Immunology 162, 30–43. doi: 10.1111/imm.13262 32935333 PMC7730020

[B56] Van Eeden C. Khan L. Osman M. S. Cohen Tervaert J. W. (2020a). Natural Killer Cell Dysfunction and Its Role in COVID-19. Int. J. Mol. Sci. 21, 6351. doi: 10.3390/ijms21176351 PMC7503862 32883007

[B57] Van Eeden C. Khan L. Osman M. S. Cohen Tervaert J. W. (2020b). Natural Killer Cell Dysfunction and Its Role in COVID-19. Int. J. Mol. Sci. 21, 6351. doi: 10.3390/ijms21176351 PMC7503862 32883007

[B58] Verdecchia P. Cavallini C. Spanevello A. Angeli F. (2020). The Pivotal Link Between ACE2 Deficiency and SARS-CoV-2 Infection. Eur. J. Internal Med. 76, 14–20. doi: 10.1016/j.ejim.2020.04.037 32336612 PMC7167588

[B59] Vivier E. Tomasello E. Baratin M. Walzer T. Ugolini S. (2008). Functions of Natural Killer Cells. Nat. Immunol. 9, 503–510. doi: 10.1038/ni1582 18425107

[B60] Wang F. Nie J. Wang H. Zhao Q. Xiong Y. Deng L.. (2020a). Characteristics of Peripheral Lymphocyte Subset Alteration in COVID-19 Pneumonia. J. Infect. Dis. 221, 1762–1769. doi: 10.1093/infdis/jiaa150 32227123 PMC7184346

[B61] Wang F. Nie J. Wang H. Zhao Q. Xiong Y. Deng L.. (2020b). Characteristics of Peripheral Lymphocyte Subset Alteration in COVID-19 Pneumonia. J. Infect. Dis. 221, 1762–1769. doi: 10.1093/infdis/jiaa150 32227123 PMC7184346

[B62] Wu Y. Ma L. Cai S. Zhuang Z. Zhao Z. Jin S.. (2021). RNA-Induced Liquid Phase Separation of SARS-CoV-2 Nucleocapsid Protein Facilitates NF-κb Hyper-Activation and Inflammation. Signal Transduction Targeted Ther. 6, 167. doi: 10.1038/s41392-021-00575-7 PMC8065320 33895773

[B63] Xiao D. Li X. Su X. Mu D. Qu Y. (2021). Could SARS-CoV-2-Induced Lung Injury be Attenuated by Vitamin D? Int. J. Infect. Dis. 102, 196–202. doi: 10.1016/j.ijid.2020.10.059 33129966 PMC7591873

[B64] Yang H. Qian H. Liu B. Wu Y. Cheng Y. Zheng X.. (2021). Triptolide Dose-Dependently Improves LPS-Induced Alveolar Hypercoagulation and Fibrinolysis Inhibition Through NF-kappaB Inactivation in ARDS Mice. BioMed. Pharmacother. 139, 111569. doi: 10.1016/j.biopha.2021.111569 34243622

[B65] Yu W. R. Fehlings M. G. (2011). Fas/FasL-Mediated Apoptosis and Inflammation are Key Features of Acute Human Spinal Cord Injury: Implications for Translational, Clinical Application. Acta Neuropathol. 122, 747–761. doi: 10.1007/s00401-011-0882-3 22038545 PMC3224722

[B66] Zarrilli G. Angerilli V. Businello G. Sbaraglia M. Traverso G. Fortarezza F.. (2021). The Immunopathological and Histological Landscape of COVID-19-Mediated Lung Injury. Int. J. Mol. Sci. 22, 974. doi: 10.3390/ijms22020974 33478107 PMC7835817

[B67] Zhang X. Zhang Y. Qiao W. Zhang J. Qi Z. (2020a). Baricitinib, a Drug With Potential Effect to Prevent SARS-COV-2 From Entering Target Cells and Control Cytokine Storm Induced by COVID-19. Int. Immunopharmacol. 86, 106749. doi: 10.1016/j.intimp.2020.106749 32645632 PMC7328558

[B68] Zhang X. Zhang Y. Qiao W. Zhang J. Qi Z. (2020b). Baricitinib, a Drug With Potential Effect to Prevent SARS-COV-2 From Entering Target Cells and Control Cytokine Storm Induced by COVID-19. Int. Immunopharmacol. 86, 106749. doi: 10.1016/j.intimp.2020.106749 32645632 PMC7328558

[B69] Zheng M. Gao Y. Wang G. Song G. Liu S. Sun D.. (2020). Functional Exhaustion of Antiviral Lymphocytes in COVID-19 Patients. Cell Mol. Immunol. 17, 533–535. doi: 10.1038/s41423-020-0402-2 32203188 PMC7091858

[B70] Zhou R. To K. K. Wong Y. C. Liu L. Zhou B. Li X.. (2020). Acute SARS-CoV-2 Infection Impairs Dendritic Cell and T Cell Responses. Immunity 53 864–877.e865. doi: 10.1016/j.immuni.2020.07.026 32791036 PMC7402670

